# Thermoelectric Materials for Textile Applications

**DOI:** 10.3390/molecules26113154

**Published:** 2021-05-25

**Authors:** Kony Chatterjee, Tushar K. Ghosh

**Affiliations:** Department of Textile Engineering, Chemistry and Science, North Carolina State University, Raleigh, NC 27695, USA; kchatte@ncsu.edu

**Keywords:** thermoelectric textiles, smart textiles, flexible thermoelectrics, carbon nanotubes, energy harvesting

## Abstract

Since prehistoric times, textiles have served an important role–providing necessary protection and comfort. Recently, the rise of electronic textiles (e-textiles) as part of the larger efforts to develop smart textiles, has paved the way for enhancing textile functionalities including sensing, energy harvesting, and active heating and cooling. Recent attention has focused on the integration of thermoelectric (TE) functionalities into textiles—making fabrics capable of either converting body heating into electricity (Seebeck effect) or conversely using electricity to provide next-to-skin heating/cooling (Peltier effect). Various TE materials have been explored, classified broadly into (i) inorganic, (ii) organic, and (iii) hybrid organic-inorganic. TE figure-of-merit (*ZT*) is commonly used to correlate Seebeck coefficient, electrical and thermal conductivity. For textiles, it is important to think of appropriate materials not just in terms of *ZT*, but also whether they are flexible, conformable, and easily processable. Commercial TEs usually compromise rigid, sometimes toxic, inorganic materials such as bismuth and lead. For textiles, organic and hybrid TE materials are more appropriate. Carbon-based TE materials have been especially attractive since graphene and carbon nanotubes have excellent transport properties with easy modifications to create TE materials with high *ZT* and textile compatibility. This review focuses on flexible TE materials and their integration into textiles.

## 1. Introduction

Cooling accounts for nearly 20% of the total electricity consumed in buildings around the world, projected to increase from 2020 terawatt-hours in 2016 to 6200 terawatt-hours in 2050 [1]. One way to combat this is by expanding the setpoint of air conditioners and heaters using personal cooling devices such as a TE cooler (TEC) [2]. A TEC is a semiconducting, solid-state heat pump operating on the Peltier effect that transfers heat from one side of the device to the other [3]. TE coolers/heaters provide the advantage of highly reliable cooling/heating with no mechanical moving parts, compact in size and light in weight, and no working fluid [4]. Additionally, they have the advantage of being powered by DC electric sources. Localized thermoregulation by wearable TE cooling devices can decrease the usage of traditional systems, thereby reducing global reliance on space heating and cooling and providing savings on energy costs [5]. Integration of TECs for on-body cooling using textiles can provide customizable thermoregulation. Textiles provide an accessible platform for the deployment of TEC devices due to the conformal and intimate contact they make with the body. Additionally, the hierarchical nature of fabrics as they progress from fiber to yarn to fabric allows the integration of TEC modules directly into the woven structure, thereby creating a more seamless fabric-based TEC device [6,7]. 

Since the discovery of the Seebeck effect (conversion of heat into electrical energy) in 1822 by Thomas Seebeck and Peltier effect (conversion of electrical energy into cooling/temperature gradient) by Jean Peltier in 1834, TE devices have been sought as solutions to make refrigerators and power generators obsolete [8,9]. The appeal to TE devices has persisted due to their potential to deliver solid-state cooling or power generation without any moving parts, toxic emissions, or loud sound during operation [10,11]. Additionally, more than 90% of the energy we use is generated by thermal processes, and conversely, heat energy is the primary form in which we waste energy [12]. Hence, implementing TE devices for thermal comfort and power generation can be an eco-friendly solution to meet global energy demands [13,14]. To realize effective TE performance, researchers focus on two key areas: (i) improving the performance of TE materials [11,15,16,17,18,19,20], and (ii) rational TE device design for performance optimization [21,22,23,24]. The dimensionless figure of merit (*ZT*) is used to express the performance of TE materials, and is expressed as $ZT=S^{2}\sigma T/\kappa$ where *S*, *σ*, *T*, and *κ* are the Seebeck coefficient, electrical conductivity, total thermal conductivity, and the absolute temperature, respectively [13,25,26]. To improve the performance of TE materials either the power factor (PF = *S*^2^*σ*) of the material has to be increased or the thermal conductivity has to be decreased using various methods such as doping, nanostructural engineering, or by developing new materials [11,27,28,29]. Many excellent reviews exploring the recent developments in TE materials and devices have also been published [11,12,25,30,31,32,33,34,35]. 

The energy and entropy transportation in TE devices is caused by the motion of charges in TE materials. Consider the case of the Peltier effect, where current flowing through a pair of n-type and p-type materials connected in series causes cooling at the junction. In this case, the electrons in the n-type material and holes in the p-type material carry heat away from the metal-semiconductor junction. A material is referred to as a hole (or electron) transporter when its ionization energy (or electron affinity) closely matches the Fermi level of the electrode material that is used to inject charges into the material [36]. Conversely, if a temperature gradient is maintained between the two ends of the n-p junction, electrons and holes diffuse to the cold side due to their higher thermal energy, thereby creating a potential difference–known as the Seebeck effect [37].

*ZT* governs the performance of TE materials, and to achieve high *ZT*, TE materials should have high *σ*, high *S* and low *κ*. The thermal conductivity, *κ* = *κ_l_* + *κ_e_*, takes into account contributions from both lattice vibrations (lattice thermal conductivity, *κ_l_* which characterizes the transport of thermal energy carried by phonons in the form of lattice vibrations) and electronic thermal conductivity (*κ_e_* = *LσT*, where *L* is the Lorenz number in the Wiedemann-Franz law) [11]. In highly doped semiconductors, S can be expressed using the Pisarenko relation [30] shown in Equation (1).

(1) S=8π2kB23eh2m*Tπ3n23

where *k_B_*, *h*, *n* and m* are the Boltzmann constant, the Planck constant, charge carrier concentration and the density of states (DOS) effective mass, respectively [35].

**Figure 1:**
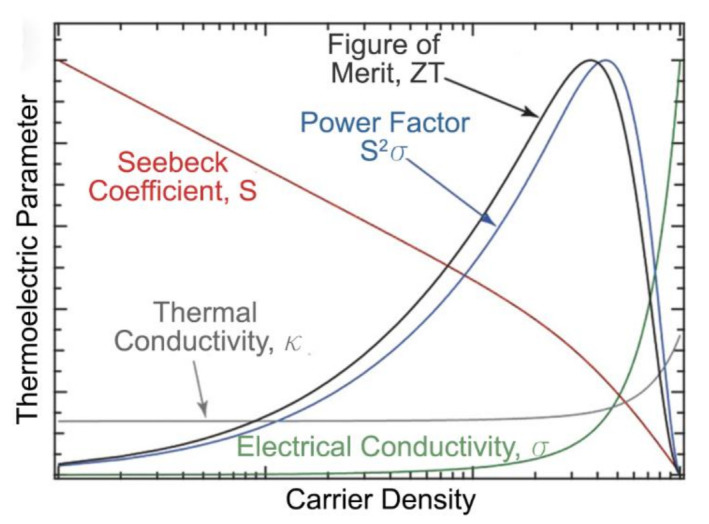
Interdependence of various TE parameters that influence *ZT* of the material, illustrating the challenge of optimizing *ZT* [33]. Data of actual semiconducting single-walled carbon nanotube (SWCNT) networks, reproduced with permissions [33]. Copyright 2018 WILEY-VCH Verlag GmbH & Co. KGaA, Weinheim.

Hence, while the task to optimize the performance of TE materials can simply be expressed as optimization of *ZT*, it is important to note that the three factors, *S*, *σ* and *κ* are intricately interlinked and are “mutually counterindicated”, as shown in Figure 1 [30,38]. This mutual counterindication can be seen in the *ZT* equation when it is rewritten with *σ* = *neµ*, where *n* is the charge carrier concentration, *e* is its charge and *µ* is its mobility. Then, *ZT* can be expressed as:

(2) $$ZT=\frac{S^{2}\sigma }{\kappa }T=\left(S^{2}n\right)\left(\frac{\mu }{\kappa }\right)eT$$

where the ratio (*µ*/*κ*) is counterindicated since defects and impurities that affect charge mobility also affect thermal conductivity and the product (*S*^2^*n*) is counterindicated because higher charge carrier concentration can lead to lower thermopower, as indicated by the Pisarenko relation in Equation (2) [30]. 

Hence, to enhance the performance of TE materials, strategies involve reducing, specifically, *κ_l_* by using methods such as strengthening the phonon scattering of materials through various nano-microstructural methods or using TE materials that have specific lattice vibrational modes that result in intrinsically low *κ_l_* values [11]. The other strategy is to enhance PF by developing new classes of materials [39,40,41,42], optimizing existing materials via doping and band engineering [43,44,45,46], and developing nanostructured materials with favorable TE properties [47,48,49,50]. In the case of semiconductors, generally TE power and electrically conductivity change in opposite directions with doping−attributed to the charge-transport theory, and hence a compromise has to be achieved between the two [25]. 

**Figure 2:**
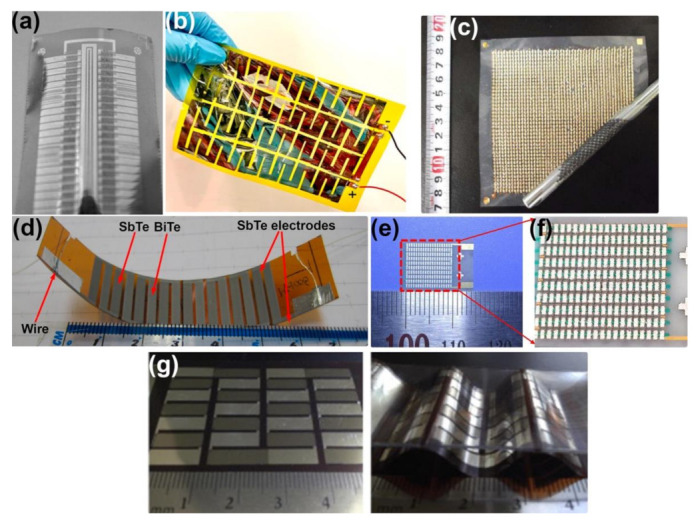
Thin film flexible TE devices. (**a**) Screen printed Sb_2_Te_3_/Bi_2_Te_3_ TE legs on a flexible polyimide substrate [52]. Reproduced with permissions [52]. Copyright 2010 Elsevier B. V. (**b**) Large-area (50 cm^2^) flexible p-SnTe and n-PbTe TEG with 32 TE pairs deposited via thermal evaporation on a polyimide substrate [51]. Reproduced with permissions [51]. Copyright 2020 Elsevier B. V. (**c**) Carbon nanotubes (CNT)/polystyrene composite p-type TE legs solution printed onto a polyethylene naphthalate film substrate [59]. Reproduced with permissions [59]. Copyright 2013 AIP Publishing. (**d**) Screen printed planar Bi_1.8_Te_3.2_/Sb_2_Te_3_ thermocouples on a polyimide substrate [68]. Reproduced with permissions [68]. Copyright 2015 Elsevier B. V. (**e**) and (**f**) BiTe and SbTe films deposited onto an AlN substrate to create 200 pairs of TE legs [69]. Reproduced with permissions [69]. Copyright 2020 Elsevier Ltd. (**g**) Corrugated thin film TE generator composed of Ag and Ni patterned onto a polyimide substrate [70]. Reproduced with permissions [70]. Copyright 2015 Elsevier Ltd.

There has been a vast amount of research conducted in the field of thin film-based flexible TE devices (FTEs) due to the fact that compared to bulk TE devices, FTEs provide the advantage of providing a conformable structure that can make intimate contact with a curved heat source (such as skin) [51,52,53,54], lower temperature processing than bulk TE materials [55,56,57,58], as well as the fact that FTEs are lightweight and less bulky than their rigid counterparts [19,20,59]. Various materials have been explored for creating FTEs such as Fan et al. developed thin film FTEs with n-type Al doped ZnO and p-type Zn-Sb to create a flexible device with a maximum power output of 246.3 µW [60], Parashchuk et al. developed p-type BiSbTe thin films on a flexible polyimide substrate with a figure of merit as high as 2.4 × 10^3^/K [61], Karthikeyan et al. used n-type PbTe and p-type SnTe to develop thin film FTEs for wearable energy harvesting with a power density of 8.4 mW/cm^2^ [51], Jiang et al. fabricated n-type Ag_2_Se films on a porous nylon membrane with a power density of 22 W/m^2^ [62], Tian et al. developed flexible organic-inorganic hybrid n-type TiS_2_/hexylamine treated superlattice structure with a power density of 2.5 W/m^2^ [57], Wan et al. also developed hybrid organic-inorganic TiS_2_ superlattices with power factor as high as their inorganic counterparts at 904 µW/mK^2^ [63]. While it is beyond the scope of this review to cover the vast literature on thin film FTEs, a number of excellent reviews which discuss both materials and configurations of thin-film FTE structures are recommended to readers (Figure 2) [10,35,64,65,66,67].

Nevertheless, in many of the cases of film-based FTEs, as shown in Figure 2, it is apparent that these are not comfortable, and wearable in the way convetional textile fabrics are. While film-based TEs can be integrated into wearable devices, it is important to understand that there is a distinction between “wearable” and “textile-based”. Since most convetional wearable textiles are composed of woven and knitted products, it is important to explore the application of FTEs when integrated into textile fibers, yarns, and fabrics. These applications have been reviewed in the past by Wang et al. [71] and Wu et al. [72] who categorized textile-based TE devices based on their structure (fiber, yarns or fabrics), and Zhang et al. [73] who focused on fiber-based TE structures, this review emphasizes the application of TE-based devices in wearable heating/cooling and the materials used to do the same. Table 1 illustrates some examples of textile-based TE devices that will be further discussed.

## 2. Flexible Thermoelectric Materials

For integration into textiles, one of the most obvious requirements for TE materials and devices is to be flexible and conformable. Being able to create conformal or FTEs not only makes the textiles comfortable but it enables better contact with the human body both for body heat harvesting to generate power and to impart TE temperature regulation via cooling or heating [35]. Additionally, bulk semiconductors pose certain limitations in terms of TE performance: the only way to reduce κ without affecting *S* or *σ* in bulk materials is by using semiconductors of high atomic weight such as Bi_2_Te_3_ and its alloys with Pb, Sn, and Sb [82]. This in turn makes these materials very expensive and their processing quite complex. Hence, thin-film TE materials can be more easily processed and be tailored for higher *ZT* than bulk materials. A variety of materials have been explored for creating FTEs, and these can be classified into three types: (1) inorganic thin film TEs [60,83,84,85], (2) organic-inorganic hybrid FTEs [63,86,87], and (3) organic FTEs–which themselves can be classified into two categories: (i) small molecules or oligomers which are processed using vacuum techniques, and (ii) polymers which are processed using wet chemistry [19,36,88,89]. Subsequent sections will explore the application of these materials in textile form factors as wearable TE devices. 

### 2.1. Inorganic Thin-Film Thermoelectric Materials

A film is considered thin as long as its surface properties are different from its bulk behavior, extending from a few micrometers to the nanometer [90]. Thin-film devices are usually prepared via deposition techniques that can be classified into either physical vapor deposition (PVD) or chemical vapor deposition (CVD). Compared to bulk alloyed materials used in state-of-the-art devices such as p-type Bi_x_Sb_2−x_Te_3−y_Se_y_ (x ≈ 0.5, y ≈ 0.12) and n-type Bi_2_(Se_y_Te_1−y_), thin-film TE materials (thickness less than 10 nm) [91] offer the advantage of being able to achieve higher *ZT* values using techniques such as quantum-confinement effects to obtain an enhanced density of states near the Fermi energy [91,92], creating superlattice (SL) structures with low *κ_l_* values [85,93], and creating heterostructures [94,95]. To understand the advantage that thin-film TEs provide over their bulk alloyed counterparts more clearly, take the example of thin films of Bi_2_Te_3_: its crystal structure’s unit cell consists of five covalently bonded monoatomic sheets along the c-axis arranged in the sequences—Te^(1)^—Bi—Te^(2)^—Bi—Te^(1)^, where ^(1)^ and ^(2)^ indicate the different bonding states of the anions [96]. Te^(1)^ and Bi are bonded via covalent and ionic bonds, whereas Te^(2)^ and Bi are bonded purely by covalent bonds. Between neighboring Te^(1)^ layers there exists a very weak van der Waals attraction. The anisotropic TE properties of thin films such as Bi_2_Te_3_ are attributed to the fact that their lattice constant along the c axis is approximately 7 times larger than that along the a and b axes [96]. Hence, Bi_2_Te_3_ thin films have electrical conductivity ~3 times higher in the ab plane compared to the c axis, and *κ_l_* value ~2 times higher in the ab plane (1.5 W/mK) compared to the c axis (0.7 W/mK) [96]. 

Various materials have been explored for use as thin-film TE materials, including those based on Bi-Te [52,83,84,97,98], Zn [60,99,100,101], Cu [102,103,104,105], and cobalt oxide [106,107,108,109] based thin films. Inorganic thin films are usually applied onto various flexible substrates using either physical vapor deposition methods such as reactive sputtering [58,104,110,111], thermal coevaporation [97,112,113] and magnetron sputtering [114,115,116], ALD [74], printing [100,117], spin coating [118], and chemical bath deposition methods [119,120,121,122]. For integration into textiles, such inorganic materials are usually deposited onto flexible, organic substrates to allow flexibility and wearability. Such wearable TE devices are usually for energy harvesting applications in the form of the Seebeck effect [74]. However, some of these substrates are unable to withstand high temperatures required for processing the inorganic TE materials, and hence free-standing thin films have also been explored. Free-standing inorganic TE thin films can be fabricated via nanostructure tailoring that involves using randomly oriented nanolaminated grains and void spaces as a substrate for the thin film, creating flexible inorganic TE thin films that can be removed from the substrate to form free-standing films [53]. Another method of creating free-standing thin films is by using carbon nanotubes (CNTs) as scaffolds to guide the deposition and growth of layered Bi_2_Te_3_ thin films that can form a hybrid free-standing structure [123]. 

**Figure 3:**
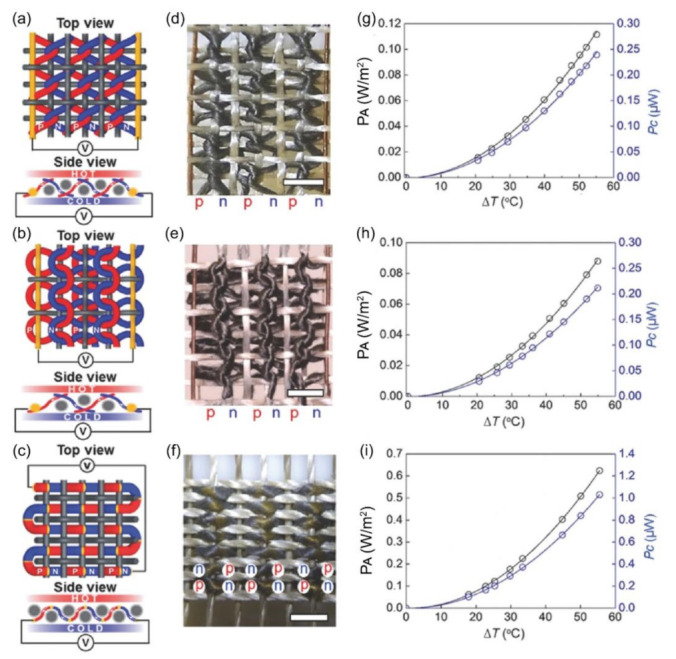
Woven-yarn TE fabrics [75]. (**a**–**f**) Illustration (**a**–**c**) and photographs (**d**–**f**) of zigzag, garter, and plain-weave TE textiles, respectively. Scale bar = 2 mm. (**g**–**i**) The output power per textile area and per TE couple as a function of applied thermal gradient (Δ*T*) for zigzag, garter, and plain-weave TE textiles, respectively. Reproduced with permissions [75]. Copyright 2016, 2016 WILEY-VCH Verlag GmbH & Co. KGaA, Weinheim.

Integration of thin-film TE into textiles has been primarily focused on creating small-scale energy harvesting devices that can power other on-body wearable devices by harvesting the body heat via the Seebeck effect [74,75]. Lee et al. fabricated thermoelectric yarns with n and p-type Bi_2_Te_3_ and Sb_2_Te_3_ coated onto aligned electrospun polyacrylonitrile (PAN) yarns using magnetron sputtering to produce a sheath-core structure with the semiconductor materials as sheath and PAN as the core [75]. Three different fabric designs—plain woven, zigzag stitched and garter stitched—were used to convert an applied thermal gradient to electrical power, harvesting heat through the thickness of the fabric rather than in the plane of the fabric, as shown in Figure 3. Additionally, fiberglass yarn coated with polytetrafluoroethylene (PTFE) was used as an insulating spacer yarn within these structures. Lee et al. observed that the plain-woven yarn with alternating n and p-type TE segments within the same yarn provided a much higher output power (0.62 W/m^2^) than those made my knitting individual n and p-type yarns together (0.11 and 0.24 W/m^2^ for zigzag and garter stitched fabrics) [75]. While this is a good use of the inherent structure of textiles to create a TE generator that can harvest power through its thickness, it is important to note that the high amount of power can only be harvested at thermal gradients (Δ*T*) greater than 50 °C [75]. Such high thermal gradients between the skin and the surroundings are seldom encountered since the average temperature of the skin ranges from the range is between 33.5–37.5 °C, and the normal habitable environment temperature ranges from 5–40 °C, thereby providing a maximum Δ*T* of ~30 K [77], beyond which humans can experience significant thermal discomfort [124,125,126]. Other implementations of coating inorganic TE materials onto textiles for TE energy harvesting have also been explored by Kim et al. who screen printed Sb_2_Te_3_ and Bi_2_Te_3_ pellets on a bendable glass textile and subsequently integrated this structure into flexible rubber sheets [76], Yadav et al. who deposited Ni-Ag thin films onto silica fiber by thermal evaporation [127], Shin et al. who screen printed Bi_0.5_Sb_1.5_Te_3_ and Bi_2_Te_2.7_Se_0.3_ inks onto glass fabrics [128], and Liang et al. who dip-coated nanocrystalline PbTe onto glass fibers [129]. In all these cases, it is apparent that the substrates used (glass, rubber, and silica) may be flexible or in a “fiber/fabric” form factor but aren’t truly suitable for use as wearable devices due to the inherent discomfort caused by such materials. Lu et al. deposited nanostructured n-type Bi_2_Te_3_ (Seebeck coefficient 36.8 µV/K) and p-type Sb_2_Te_3_ (Seebeck coefficient 110.8 µV/K) onto commercially available silk fabric to form ~300 µm thick TE columns for use as human body heat harvesters [77]. They measured the performance of their device in the Δ*T* range of 5 to 35 K, reporting an output power ranging from ~2–18 nW. While this technique did use a textile substrate, the overall TE performance of the device was quite low due to the use of a liquid adhesive binder, as well as the contact resistance between the two sides of the fabric where the n and p-type materials are deposited [77]. 

**Figure 4:**
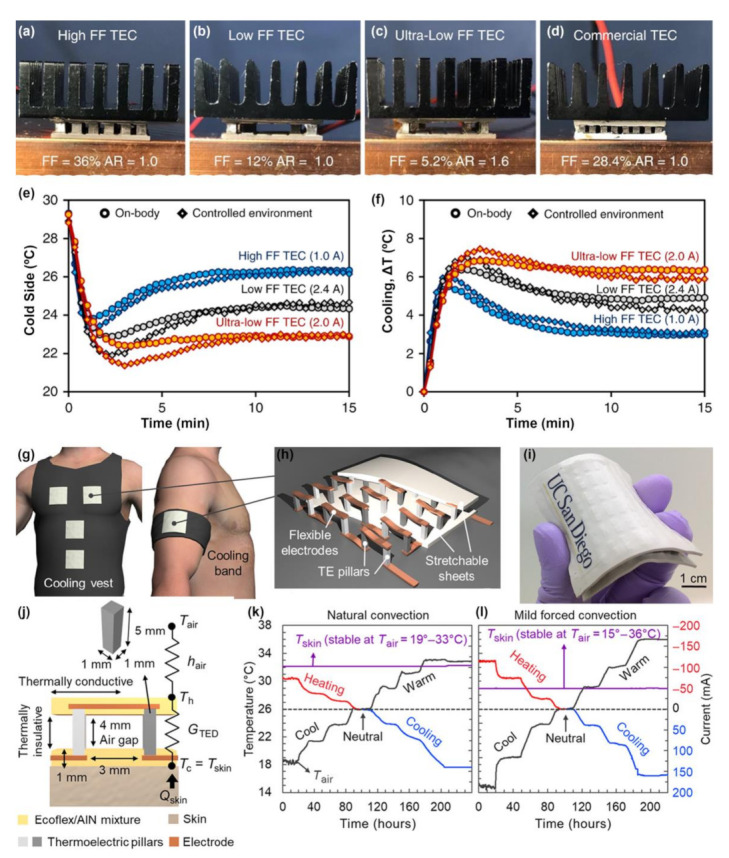
Wearable TEC for localized cooling [5]. (**a**–**d**) TEC modules fabricated with commercial p- and n-type Bi-Te with (**a**) high fill factor (FF) (FF = 36%, aspect ratio, AR = 1.0), (**b**) low FF (FF = 12%, AR = 1.0), (**c**) ultra-low FF (FF = 5.2%, AR = 1.6), and (**d**) commercial TEC module (FF = 28.4%, AR = 1.0). The black heat sink is anodized aluminum. (**e**,**f**) Show transient temperature data from cooling the human body and in controlled environments, for the various TECs. The optimal currents are 1 A for high FF TEC (blue), 2.4 A for low FF TEC (black), and 2 A for ultra-low FF TEC (red). Ultra-low FF TEC generates the lowest cold side temperature and has the highest cooling. Reproduced with permissions [5]. Copyright 2019, Creative Commons Attribution 4.0 International License (CC-BY-4.0). Flexible TED for on-body cooling [130]. (**g**) Wearable TE device (TED) integrated into a vest and arm band. (**h**) TED consists of alloy pillars connected with flexible copper electrodes and silicone sheets. (**i**) A 5 cm × 5 cm fabricated TED. (**j**) TED design with low thermal conduction inside and high thermal conduction within the silicone sheets enables cooling without the use of a heat sink. *T*_air_ = ambient temperature, *h*_air_ = heat transfer coefficient between TED and air, *T*_h_ = temperature at the top of TED, *G*_TED_ = thermal conductance of TED, *T*_c_ = *T*_skin_= temperature at the bottom of TED, *Q*_skin_ = human metabolic heat. (**k**) Thermal regulation by TED under natural convection shows that the surface temperature of the silicone layer remained 26 °C in temperature range of 19–33 °C. (**l**) With forced convection of 5 km h^−1^ the ambient temperature range broadens to 15–36 °C. Reproduced with permissions [130]. Copyright 2019, Creative Commons Attribution NonCommercial License 4.0 (CC-BY-NC).

While research in TECs is scant compared to thermoelectric generators (TEGs), the interest in achieving TE heating and cooling for human thermal comfort has been growing in recent years. Moreover, there is only a limited amount of temperature gradient that the human body can provide for TEGs, and coupled with their low *ZT* in many cases, TEGs are only capable of producing a few microwatts or nanowatts of power [130]. Lee et al. and Park et al. have demonstrated FTECs using inorganic, rigid Bi-Te p and n-type modules arranged in a mat-like fashion connected with wires and containing a flexible heat sink composed of solid-state silica gel mixed with hydrogel [131,132,133]. These devices were able to cool the skin by a temperature drop of 3.8 K with a cooling power of 30 mW cm^−2^ [133], with an improvement to 4.4 K and cooling power of 33.5 mW cm^−2^ and cooling power of 5.4 K and 48.3 mW cm^−2^ when the contact resistances in the devices were reduced by using flexible printed circuit boards (FPCB) [132] and liquid metal electrodes [132], respectively. Kishore et al. also developed high-performance wearable coolers which were able to cool the skin by a temperature drop of 8.2 °C below room temperature, as shown in Figure 4a–f [5]. They achieved this by using commercially available n and p-type Bi-Te materials to fabricate a rigid TEC module with an aluminum heat sink. In all these instances, it is apparent that current research on TECs involves the use of rigid semiconductor materials. This can cause an obvious mismatch between the softness of the skin and the rigidity of the TEC, creating discomfort for the wearer. Hong et al. demonstrated a flexible TEC without the use of rigid or bulky heat sinks by sandwiching inorganic TE pillars between two layers of stretchable elastomers embedded with aluminum nitride (AlN) microparticles that enhanced the sheets’ lateral thermal conductivity, as shown in Figure 4g–l [130]. This design creates a large air gap between the elastomer sheets, resulting in small thermal conductance between the hot and cold side of the TEC. In this way, they were able to ensure that the heat pumped from the cold side of the device and the expectant Joule heating in the device would dissipate into the air rather than back towards the skin. Additionally, the AlN embedded elastomer sheets ensured that the heat would spread uniformly throughout the sheets, enabling better heat dissipation. In this way, Hoang et al. were able to create a TEC with a long-lasting cooling effect of >8 h and a large active cooling effect >10 °C [130]. 

However, in all these instances, it is apparent that efficient TECs could only be fabricated using inorganic TE materials. It is not given that material suitable for TEGs can be easily translated into a Peltier cooler. In the latter case, the TEC must be able to efficiently dissipate the waste heat generated from the cold side pumping heat away from the skin. Additionally, it must overcome both parasitic heating due to heat conduction from the hot side, as well as resistive heating within the device. Hence, the device and material requirements for a TEC are much different from a TEG. While currently, TECs are using rigid materials, there is great potential in the development of new materials for both TECs and heat sinks that can create more efficient cooling. 

While inorganic thin film TE materials are capable of producing high *ZT* with low *κ_l_*, their applications are limited mainly due to the low abundance of tellurium, which is one of the rarest elements in the Earth’s crust [74]. Additionally, the interface formed between inflexible inorganic TE films and flexible fabrics are likely to be weak and cause discomfort in next-to-skin applications. The addition of a higher amount of TE material for better heating/cooling performance would also result in added bulk to the fabric, and hence other materials more conducive for wearable applications have also been explored. 

### 2.2. Organic Thermoelectric Materials

Organic TEs (OTEs) provide considerable advantages over inorganic TE materials due to their inexpensive and scalable processing methods unlike inorganic materials which need complex vacuum processing methods, have the potential for heavy metal pollution, and have low abundances [34]. The lightweight and flexible nature of OTEs enables better integration into textiles; inorganic thin-film semiconductors usually end up being brittle and bulky [73,89]. OTEs also generally have thermal conductivity below 1 W/mK and have tunable molecular chemistry through doping that can enhance their TE performance, however, they are sometimes limited by their low power factors (PF = *S*^2^*σ*), with their PF being 2–3 orders of magnitude lower than those of inorganic TE materials [73,134,135]. Enhancement of OTE performance is focused more on optimizing their PFs rather than optimizing their thermal conductivity, unlike inorganic TEs where the focus is on reducing the thermal conductivity. 

OTEs can be further classified broadly into two categories: (i) small molecules or oligomers which are usually processed in a vacuum, and (ii) polymers which are usually processed by wet chemical techniques [36]. In OTE polymers, the interaction between the polymer unit cells leads to the formation of electron bands; the highest occupied electronic level consists of the valence band (VB) (also approximated as highest occupied molecular orbital or HOMO or oxidation potential) and the lowest unoccupied level consists of the conduction band (CB) (also approximated as lowest unoccupied molecular orbital or LUMO or reduction potential), as shown in Fiugre 5 [136]. The width between the VB and CB is known as the forbidden band or energy bandgap (E_g_) whose value ranges between 1–3 eV for OTE materials [34,137]. Due to this relatively wide bandgap, many organic semiconductors need to be doped to increase their electrical conductivity. Doping is used in OTEs to either generate mobile carriers by donating electrons to the LUMO state (n-type doping) or remove electrons from the HOMO state (p-type doping). However, n-type doping is more difficult to achieve in OTEs because the HOMO level of the dopant has to be energetically above the LUMO level of the polymer being doped, making such materials unstable when exposed to oxygen [138]. Moreover, the electrical conductivity of these materials is affected both by introduction of carriers and their structural order ranging from the molecular scale to the macroscale [137].

**Figure 5:**
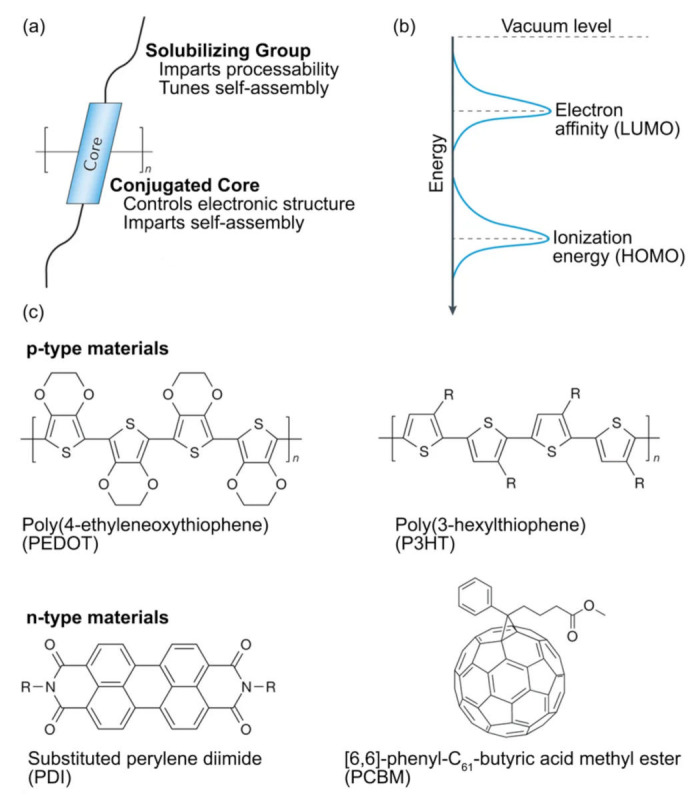
Organic TE materials and their structures. (**a**) Polymeric semiconductors have π-conjugated cores that enable charge transport and side chains that can impart solution processability, charge carrier creation, and molecular assembly. (**b**) Ionization energy (HOMO) and electron affinity (LUMO) of polymeric semiconductors can be tuned via their molecular design. (**c**) Molecular structures of high-performance p and n-type OTEs. Adapted from Russ et al. [34] copyright 2016, The Authors.

The majority of OTE materials are based on conductive polymers which include conjugated and some coordination polymers. The electronic structure of the π conjugated polymers originates from the sp^2^p_z_ hybridized wavefunctions of the carbon atoms in the repeat units of the polymer, leading to one unpaired electron per carbon atom; this creates an electron delocalization that enables charge mobility along the polymer backbone chain, as shown in Figure 5 [134,139]. Conjugated polymer repeat units in the backbone consist of both covalent *σ* bonds and delocalized alternating π-bonds, with the conjugated repeat units strongly electronically coupled along the backbone of the polymer, but weakly coupled between stacked chains of the polymer [140,141]. Since the discovery of doped polyacetylene in the late 1970s [142], conjugated polymers such as polyactelyenes [143,144], polypyrroles [145,146], polyanilines (PANI) [147,148], polthiophenes [149,150], polycarbazoles [151,152], and their derivatives have been widely used for various TE applications [153]. In terms of textile applications, the state of the art OTE is poly(3,4-ethylenedioxythiophene) (PEDOT) and its derivative poly(styrenesulfonic) (PSS) acid-doped PEDOT (PEDOT:PSS) with a *ZT* of 0.42 [135,154,155,156,157,158,159], with composites of polyaniline (PANI) [80,160,161] also being explored. While PEDOT:PSS has better TE properties compared to PANI due to the former’s bipolaron network compared to PANI’s polaron network [73], it is still limited by the fact that it forms brittle films on textile substrates which cannot withstand the various stresses and strains textiles experience.

Most reported OTEs are intrinsically p-type because the electron affinity of organic polymers is usually low, and hence n-type behavior is difficult to obtain without doping [73]. Reported n-type OTEs include fullerenes [162,163], organometallic derivatives [88,164], and other small molecules [165,166]. However, fullerenes and organometallic derivatives are not solution-processable which severely limits their usability in textile applications; small molecules such as poly{N,N′-bis(2-octyl-dodecyl)-1,4,5,8-napthalenedicarboximide-2,6-diyl]-alt-5,5′-(2,2′-bithiophene)} (P(NDIOD-T2) [166] have complex fabrication processes, low TE performance and cannot be used at a large scale [120]. To combat some of these issues, Shi et al. developed three solution-processible n-type polymers: benzodifurandione-based poly(p-phenylene vinylene) (BDPPV), chlorine-BDPPV (ClBDPPV) and fluorine-BDPPV (FBDPPV) which showed high electrical conductivities of up to 14 S/cm and power factors up to 28 µW/mK^2^ when mixed with the n-type dopant ((4-(1,3-dimethyl-2,3-dihydro-1H-benzoimidazol-2-yl)phenyl)dimethylamine) (N-DMBI) [167]. N-DMBI is often used as a dopant for n-type organic semiconductors such as [6,6]-phenyl C_61_ butyric acid methyl ester (PCBM) [168] and P(NDIOD-T2) [166] due to its good chemical and air stability and efficient doping properties. More recently, Serrano-Claumarchirant et al. demonstrated n-type behavior for the first time in PEDOT thin films by treating it with cationic surfactant dodecyltrimethylammonium bromide (DTAB), reaching a maximum value of ~−21 µV/K, with a 3 order of magnitude decrease in electrical conductivity compared to p-type PEDOT films [169]. This decrease in electrical conductivity is clearly undesirable for TE performance.

**Figure 6:**
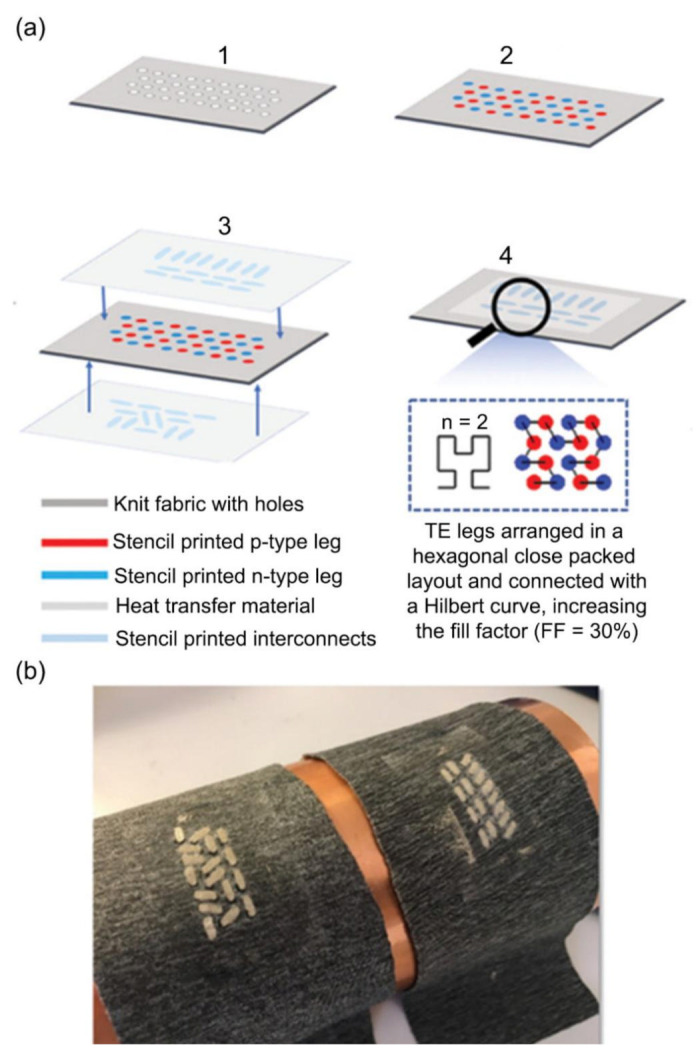
Polymer TEG for human body heat harvesting [135]. (**a**) Fabrication process for textile integrated TEG: 1. Burning holes through the knitted fabric, 2. Filling the holes by stencil printing p- (red) and n-type (blue) materials on both sides of the fabric, 3. Silver interconnects (light blue) are printed onto a heat transfer membrane (light gray) on both sides of the device, 4. Heat pressing interconnects onto both sides of the device using the heat transfer membrane. (**b**) Wearable TEG integrated into knitted fabric consisting of 32 p- and n-type legs. Reproduced with permissions [135]. Copyright 2019 WILEY-VCH Verlag GmbH & Co. KGaA, Weinheim.

Other developments in solution processible n-type OTEs include metal-coordination compounds, specifically metal-dithiolene coordination polymers, consisting of bridging ethenetetrathiolate ligands and nickel metal centers (nickel ethenetetrathiolate, NiETT) (poly[Na(NiETT)]) have resulted in higher performing n-type OTEs with electrical conductivity of 44 S/cm and power factor of 20 µW/mK^2^ [170,171]. Elmoughni et al. demonstrated a textile-integrated TE generator consisting of both p and n-type organic semiconductors: PEDOT:PSS and poly[Na(NiETT)] respectively, stencil printed onto a knitted polyester fabric, as shown in Figure 6 [135]. The unique deposition of the n and p-type legs in a hexagonal closed-packed layout helped achieve higher fill factor (~91%), allowing for higher power density due to lower interconnect resistances [172]. At Δ*T* = 3 K, such a device consisting of 32-legs and 864-legs was able to provide a maximum open circuit voltage of 3 mV and 47 mV. While this is an innovative application of fractal design to enhance the density of the TE legs, the device performance was still limited by the fact that OTEs have low power factors compared to their inorganic and hybrid counterparts and provide imperfect contacts between the organic p and n-type legs and the inorganic interconnects used to connect them in a TE device [135]. For this reason, organic composites consisting of polymeric semiconductors combined with nanomaterials such as CNTs [55,173,174,175], graphene [176,177,178,179], and reduced graphene oxide (rGO) [180,181] have also been explored to create OTEs with enhanced n and p-type performance. 

#### Carbon-Based Thermoelectric Materials

Graphene consists of a two-dimensional (2D) sheet of covalently bonded carbon atoms, forming the basis for both 3D graphite and 1D CNTs [182]. Due to its unique electrical [183,184], mechanical [182], thermal [185], and optical properties [186], graphene is has been studied extensively for various flexible electronics applications [187,188,189,190]. Although its high thermal conductivity makes it more applicable for passive cooling rather than TE applications [191,192], researchers have predicted that the TE performance of graphene can be improved by patterning it into quasi 1D graphene nanoribbons (GNRs) which have lower thermal conductivity than graphene [193,194,195]. GNRs can be viewed as unrolled CNTs, with widths as low as a few nanometers [196]. Zheng et al. found that at such small scales, the quantum confinement effect enables an increase in *ZT* with decreasing ribbon width, and by optimizing the doping level a room temperature *ZT* of 0.6 could theoretically be obtained [197], Ouyang and Guo found through theoretical modeling that the thermopower of GNRs (in the order of mV/K) is much larger than that of graphene (<100 µV/K) due to the existence of a bandgap in semiconducting GNR compared to gapless 2D graphene [195]. Other theoretical methods proposed to reduce the thermal conductivity of GNR without compromising its electrical performance include edge disorders [198], edge passivation [199], doping with carbon isotopes [200], mechanical straining [201], superlattice structures [202], nanoporous GNRs and defect engineering [203,204], and Antidot lattices [205]. However, GNRs are microscopic forms of TE graphene materials, and hence composites of conducting polymers and graphene are better suited for macroscopic applications, like those in textiles. 

In terms of film-based applications of graphene and rGO for TE devices, researchers report a significant enhancement in the TE performance of intrinsically conducting polymers when combined with graphene or rGO [177,178,206,207,208,209,210,211,212,213,214,215]. Park et al. prepared a hybrid PEDOT:PSS/graphene film using rapid thermal chemical vapor deposition (RTCVD) of graphene followed by spin coating of PEDOT:PSS to create TE films with conductivity of 1090 S/cm and power factor of 57.9 µW/mK^2^ attributing the enhancement of TE properties to the π-π stacking interaction between graphene and PEDOT:PSS [216], Xiong et al. made films of PEDOT:PSS and graphene nanocomposites, reaching a maximum electrical conductivity of 1250 S/cm (1 wt.% graphene) and optimized power factor of 38.6 µW/mK^2^ (3 wt.% graphene) [217], Xu et al. proposed three different methods—spin-coating and liquid layer polymerization, spin coating and vapor phase polymerization, and in-situ polymerization and then ethylene glycol post-treatment, to make PEDOT/rGO nanocomposites, all of which showed enhanced the TE performance of neat PEDOT with a maximum power factor of 14.2 µW/mK^2^ [215], Han et al. grew polypyrrole coatings on both sides of rGO nanosheets via template-directed in situ polymerization to create rGO/PPy composites with power factor up to 3.01 µW/mK^2^ [214]. However, these nanocomposites were only used to demonstrate film-type applications which are flexible but not necessarily in any textile form factor, thereby limiting their wearability [177,207,209]. For example, Zhang et al. used to roll-to-roll printing of PEDOT:PSS and nitrogen-doped graphene inks to continuously create large-area TE devices for energy harvesting on a plasma-treated plastic film [207], whereas Xiang and Drzal folded the PANI-graphene nanocomposite film (termed as graphene/PANI paper) into an accordion design to create a flexible TE device [177], however they do not demonstrate any practical wearability akin to a woven, knitted or nonwoven fabric design. Nevertheless, this is still important research in being able to understand which materials should be further investigated for textile-based TE applications due to their enhanced PF and electrical conductivity. 

Ma et al. studied the TE properties of macroscopic graphene fibers and the dependence of their thermal conductivity, electrical conductivity, and Seebeck coefficient on temperature [78]. They noted that as the temperature goes from 80 to 290 K, the thermal conductivity increases and then decreases, electrical conductivity increases, and Seebeck coefficient changes from positive (hole dominant) to negative (electron dominant) [78]. They were further able to enhance the power factor and figure of merit of the graphene fibers to 624 µW/mK^2^ and 2.76 × 10^−3^ respectively, using bromine doping which improves phonon scattering by introducing defects, thereby decreasing the thermal conductivity [218]. Electrical conductivity and Seebeck coefficient both increased due to the lowering of Fermi levels; electrons drained towards the highly electronegative Br sites, increasing the density of holes at the top of the valence band resulting in a positive Seebeck coefficient and increased electrical conductivity [218]. This research is promising since the graphene fibers have good TE properties at low temperatures, enabling their applicability in room-temperature applications and their fiber form enables more seamless integration into textiles. However, the fiber fabrication method requires high-temperature annealing (2800 °C) with the need for vacuum processing and materials such as liquid bromine and liquid nitrogen [218] which can prove to be time-consuming and expensive.

**Figure 7:**
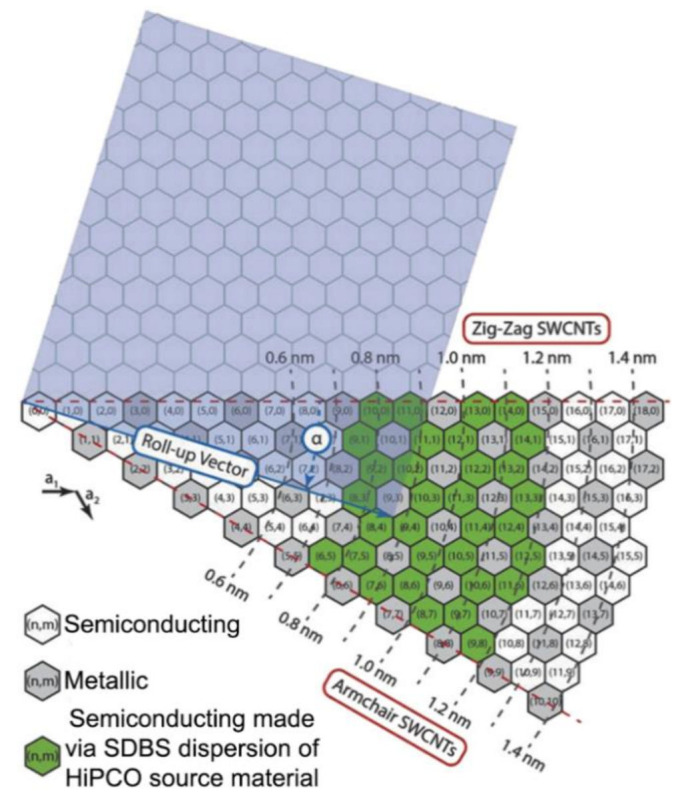
Part of a graphene sheet with chiral indices (n,m) corresponding to the SWCNT formed by rolling the sheet from (0,0) to (n,m) along the highlighted roll-up vector to form a cylinder [33]. Rolling up along the shaded blue area (indicated as a roll-up vector) forms a (9,4) SWCNT cylinder. White hexagons correspond to chiral indices that form semiconducting SWCNTs, gray hexagons to chiral indices that form metallic SWCNTs, and green hexagons to chiral indices that form semiconducting SWCNTs present in a typical batch of commercial sodium dodecylbenzenesulfonate (SDBS) dispersed SWCNTs produced by the high-pressure carbon monoxide (HiPCO) process. Reproduced with permission [33]. Copyright 2018, WILEY-VCH Verlag GmbH & Co. KGaA, Weinheim.

CNTs are attractive TE materials due to their remarkable electronic and atomic properties, enabling the nanotube to be either semiconducting or metallic, depending on the chirality indices (n, m) of the nanotubes and their diameter [219,220]. Chirality indices for SWCNTs can be understood as the roll-up vector of the graphene sheet from which it is made, as shown in Figure 7 [33]. Of all possible (n,m) combinations of nanotubes, about two-thirds are predicted to be semiconducting [221]. 

Undoped semiconducting CNTs can be thought of as being adventitiously doped by atmospheric oxygen, and hence behave as p-type semiconductors, with Seebeck coefficients measured in the range of 24–100 µV/K [222,223] and electrical conductivities ranging from 35–3200 S/cm [223,224] for such O_2_ doped SWCNTs. CNTs also present certain advantages when it comes to doping them to form n-type TE materials when compared to conducting polymers: they are porous and have high surface areas (in the case of SWCNTs) which can result in more accessible sites for redox molecules to adsorb to, the CNT network can be immersed in various dopant-containing solutions without causing a change in the morphology of the CNTs themselves, and finally, the surface of CNTs is sensitive enough to redox moieties that no covalent bonds are needed to be formed to create large changes in carrier density—just the phenomenon of physisorption can achieve efficient doping [33]. Hence, CNTs provide a facile manner of creating TE materials without complex processing steps as observed in other organic TE materials [20]. However, even with these advantages, it has proved to be difficult to achieve air-stable n-type doping in CNTs, since the electron density decreases rapidly due to O_2_ and H_2_O adsorption [224]. Nevertheless, different amine and phosphine-based dopants have been studied for n-type doping of CNTs, as demonstrated by Nooguchi et al. who demonstrated eighteen different dopants that enabled air stable n-type doping of SWCNT films [225]. Commonly used polymers for easily creating solution-processable and air-stable n-type CNTs include polyethyleneimine (PEI) [6,81,226], polyvinyl pyrrolidone (PVP) [7,227], polyvinyl alcohol (PVA) [228], poly(vinylidene fluoride) (PVDF) [229], PEG [230], and poly(3-hexylthiophene) (P3HT) [231]. It is important to note that the use of large band-gap insulating polymers such as PEI to create n-type doped CNTs should not be seen as charge injection but rather as surface modification that creates an intrinsic molecular dipole moment and a charge transfer interaction with the CNT surface, thereby leading to a decrease in work function and hence n-type behavior [232].

**Figure 8:**
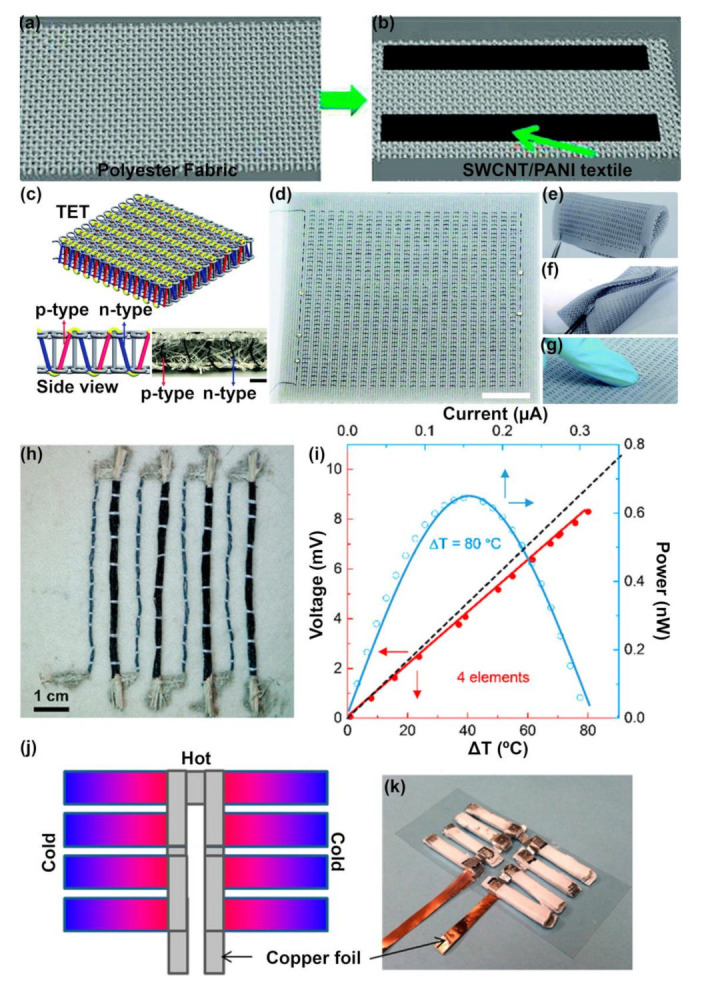
CNT-based TE devices integrated into textiles. (**a**) Polyester fabric that is coated with SWCNT/PANI composite in (**b**) to create a TE generator [77]. Reproduced with permissions [77]. Copyright 2016, The Royal Society of Chemistry. (**c**–**g**) Warp-knitted spacer fabric as TE textile (TET) with (**c**) showing the cross-section of the TET (scale bar = 2 mm), (**d**) 8 cm × 9.3 cm TET fabric (scale bar = 2 cm), (**e**) bending, (**f**) twisting, (**g**) compressing the TET fabric [6]. Reproduced with permissions [6]. Copyright 2020, The Royal Society of Chemistry. (**h**) Textile TE device with 4 p-n pairs, composed of n-type coated PET yarns (11 yarns/leg) and p-type dyed silk yarns (2 yarns/leg) with its output voltage and power generated as a function of temperature difference shown in (**i**) [7]. Reproduced with permissions [7]. Copyright 2018, American Chemical Society. (**j**) Design of a CNT-based TE module with (**k**) showing the device with 72 TE pairs. Reproduced with permissions [78]. Reproduced with permissions [78]. Copyright 2014, American Chemical Society.

For integration into textiles, CNT-based materials have been used for energy harvesting combined with other polymers to form composites [7,81,226,229,233,234], including intrinsically conductive polymers such as PANI [80] and PEDOT:PSS [6,19] for enhanced TE performance, which are illustrated in Figure 8. At the fiber/yarn level, Ryan et al. developed a TE generator composed of commercial PET sewing threads coated with multiwalled CNTs (MWCNTs)/PVP (n-type) and PEDOT:PSS dyed silk yarns (p-type) [7]. Here, the n-type yarns had a conductivity of 1 S/cm and Seebeck coefficient of −14 µV/K. Using 38 n/p yarns, they were able to produce an open-circuit voltage of 143 mV when exposed to a temperature gradient of 116 °C [7]. Zheng et al. developed MWCNT yarns coated with PEDOT:PSS (p-type) and PEI (n-type) to form three-dimensional TE textiles (TETs) for out-of-plane TE power generation [6]. The PEDOT:PSS/CNT composite yarn had an average Seebeck coefficient of 70.1 µV/K, electrical conductivity of 1043.5 S/cm and power factor of 512.8 µW/mK^2^, whereas the PEI/CNT yarn had an average Seebeck coefficient of −68.7 µV/K, the electrical conductivity of 1408.3 S/cm and power factor of 667.8 µW/mK^2^ [6]. Zheng et al. reported that the work function of the PEI/CNTs (~4.15 eV) is smaller than that of the pristine CNTs (~4.36 eV) even though they have similar band gaps. This was attributed to the dipole moments arising from both the ethylamine molecule from PEI as well as the ethylamine/CNT interface dipole as the PEI is physisorbed onto the outer layers of the MWCNTs [232]. These yarns were then knitted into a 3D fabric with a spacer in between, creating the TET with a maximum power output of 51.5 mW/m^2^ at an applied temperature gradient of 47.5 K [6]. At the fabric level, Yu et al. used vacuum filtration to deposit SWCNTs of p-type and n-type (doped with PEI, NaBH_4_, and a combination of the two) onto a PTFE membrane to obtain maximum PFs of 12.1 µW/mK^2^ (*S* = 22 µV/K, *σ* ~ 2.5 × 10^4^ S/m) and 32.49 µW/mK^2^ (*S* = −57 µV/K, *σ* ~ 1 × 10^4^ S/m) for the pristine p-type SWCNT and PEI-doped n-type SWCNT membranes, respectively [226]. Kim et al. also used a similar materials system consisting of SWCNTs deposited onto PTFE and doped with PEI and/or NaBH_4_ for n-type performance and reported PFs of 103.5 µW/mK^2^ (*S* = −97 µV/K, *σ* = 1.1 × 10^4^ S/m) for p-type pristine SWCNT membranes and 38 µW/mK^2^ (*S* = −86 µV/K, *σ* = 5200 S/m) for n-type PEI + NaBH_4_ doped membranes [81]. While these works refer to PTFE membranes as fabrics, such membrane-type materials are not suitable for use as fabrics in their traditional applications for wearable systems [229,235]. A crucial limitation of some of these works are that they measure the transport properties of such films in two different directions i.e., the Seebeck coefficient and electrical conductivities are measured in the plane of the film, whereas the thermal conductivities are measured out of plane or in some cases thermal conductivities are not reported at all [7,81,229,233]. This does not provide a true representation of the *ZT* of the TE materials, crucial for determining overall performance. While Zhou et al. [27] and Zheng et al. [6] have used the self-heating 3ω method to estimate the thermal conductivity of their n and p-type CNT legs, they large values (18 W/mK for n-type SWCNT/PEI film and 24 W/mK for p-type SWCNT film [27], and 35 W/mK for p and n-type MWCNT and MWCNT/PEI yarns [6], respectively) which depress their *ZT* further. Accurately measuring the transport properties of CNTs can be challenging, and their high thermal conductivities can be a problem when it comes to TE applications despite their large PFs.

While CNTs are promising, they still possess high thermal conductivities, and hence this can reduce their *ZT* performance. A new approach to realizing TE materials is by integrating organic and inorganic materials together to create composites where the synergistic effects of their constituents can create materials with much higher *ZT*s. The next section will explore these materials.

### 2.3. Organic-Inorganic Hybrid Thermoelectric Materials

Organic-inorganic hybrid TE materials (OInTEs) present the opportunity to take advantage of both the low thermal conductivity of TE polymers and the high *ZT*s of inorganic TE materials to obtain OInTEs with maximized *ZT*s [236]. Additionally, TE materials like Bi_2_Te_3_ suffer from the lack of flexibility, and hence combining them with TE polymers can enable flexible TE devices [237]. Moreover, by combining organic and inorganic materials, interesting interfacial transport properties arise in the resultant hybrid TE material, resulting in energy filtering and phonon scattering at the nanoscale, thereby providing an enhanced *ZT* [238]. The most commonly used ICPs include PANI, PEDOT (and PEDOT:PSS) and polythiophene (PTH) [239], which are used to create OInTEs such as PANI mixed with various metals and their oxides including Bi [240], NaFe_4_P_12_ [241], V_2_O_5_ [242], Bi_2_Te_3_ and its alloys [243,244,245] and PbTe [246], PEDOT with Te nanorods [247], Ca_3_Co_4_O_9_ [248], Au nanoparticles [249] and Bi_2_Te_3_ [250,251], and PTH with Bi_2_Te_3_ [252,253]. Since PANI is stable and has a high electrical conductivity, it can be combined easily with inorganic TE materials mainly via physical mixing of dry powders of PANI and inorganic materials such as Bi_0.5_Sb_1.5_Te_3_ [245], solution mixing [254], in situ oxidative polymerization to insert PANI in V_2_O_5_.*n*H_2_O to form a xerogel [242], and in situ interfacial polymerization to form PbTe-PANI composite nanostructures [246]. While PEDOT itself is not particularly soluble and hence limited in terms of solution processability, when emulsified with PSS to form PEDOT:PSS it has been combined with inorganic materials using in situ synthesis combined with drop casting [255], solution mixing of PEDOT:PSS with Bi_2_Te_3_ particles [250], and direct hybridization with Au nanoparticles [256]. Although PTH has low solubility and electrical conductivity, Du et al. used a two-step process of preparing Bi_2_Te_3_ and PTH using hydrothermal synthesis and oxidative polymerization respectively and then pressing them together to form a TE film [253]. While most of these studies report on composite films, Karttunen et al. demonstrated TE fabrics made via atomic and molecular layer deposition (ALD and MLD) of ZnO and ZnO-C_6_H_4_-OZn (ZnO-organic) superlattice materials onto cotton fabric [74]. These were advantageous since they could be fabricated directly onto the textile substrate, and showed power factors ranging from 7.4 to 137 W/cm.K^2^. 

While the integration of inorganic and organic TE materials to produce hybrid materials with high *ZT*s is a promising avenue, it still faces many challenges. Many of the fabrication methods require high temperatures, long processing times, and high cost of processing. Moreover, the challenge of combining rigid inorganic small molecules efficiently with polymers is a challenge [73]. Nevertheless, with the fabrication of nanowire-based OInTEs, there may be some applicability in the future for such materials into textile fiber-like form factors [257]. 

## 3. Conclusions and Future Outlook

There remains a tremendous opportunity to develop fabric-integrated, soft and flexible, on-body, seamless Peltier heating/cooling devices. This review provides an overview of the various materials and devices that have been used to integrate TE elements into textiles in the form of TEGs or TECs. While there is a lot of research on the integration of TEGs into textiles, the field of fabric-based TECs is still emerging. This translates into a good opportunity for the integration of TECs at the fiber or yarn level to create a more seamless thermal comfort experience. However, TECs require heat sinks or some method of waste heat management which may not always be the easiest to deploy. They also often have the drawback of low efficiency of organic TE materials that limits how much cooling can be achieved. Developing a personal thermal comfort system seamlessly integrated into textiles that do not need wearer intervention and can provide on-demand heating and cooling is the holy grail of research scientists working on textiles and comfort. From the review, TECs are one avenue of achieving this and there is a tremendous opportunity in the development of flexible, conformable, and high *ZT* TE materials, as well as integration of these into fabric geometries such that they do not sacrifice the inherent strength, flexibility and comfort associate with textile fabric. A systematic understanding of exactly which fabric architecture should be used to obtain the maximum cooling without sacrificing the inherent nature of textiles i.e., comfort, flexibility, and strength is needed. 

Even a 1 °C expansion of the thermostat set point of air conditioners used in the buildings in the US can result in a 7–15% increase in energy savings [258], and hence TECs do not have to provide excessive amounts of heating/cooling at this stage. Additionally, humans can perceive temperature changes of 0.02–0.07 °C of cooling, and 0.03–0.09 °C of warming pulses. The rate of temperature change is also important, with humans capable of detecting temperature changes if they happen more rapidly such as at 0.1 °C/s compared to 0.5 °C/min. Hence, lower-efficiency materials can still be used for this purpose. It is important to note that the *ZT* of a TE material, alone, is not enough to describe some of the qualitative requirements from materials that are to be used for fabric-based cooling—other characteristics of importance are room temperature and solution processability, stability of performance over time, flexibility and conformability, scalability and low cost. These qualities are not captured in *ZT* but are crucial to the creation of the next generation of TECs. 

With the growing need for thermal comfort in the backdrop of global temperature rise due to climate change [1,259], it is important to intervene at a disruptive level to be able to bring about real change to the imminent global energy crisis that growing populations, rising incomes, and greater built environments will have in the coming years. On-body thermal comfort systems can be that change and thermoelectric elements integrated at the constituent level of textile fabrics are a novel approach to achieving this.

## Figures and Tables

**Table 1:** Various TE materials used for textile-based TE devices and their performance.

Material Used [Ref.]	Textile	Preparation Method	TE Performance
ZnO–C_6_H_4_O–Zn organic superlattice [74]	Cotton fabric	ALD ^a^/MLD ^b^	Highest PF = 137 × 10^−7^ W/cm.K^2^
n- and p-type Bi_2_Te_3_ and Sb_2_Te_3_, respectively [75]	PAN ^c^ nanofibers woven and knitted into fabric	Sputter coating BiTe and SbTe onto PAN nanofibers	Power output = 0.62 W/m^2^ for plain weave, 0.11 W/m^2^ for knitted
Bi_2_Te_3_ (n-type) and Sb_2_Te_3_ (p-type) [76]	Glass fabric	Screen printing	Open-circuit output voltage = 2.9 mV, output power = 3 μW at temperature 15 °C for 11 TE pairs
Nanostructured Bi_2_Te_3_ and Sb_2_Te_3_ [77]	Silk fabric	Solution deposition	Maximum voltage ∼10 mV, power output ∼15 nW
Graphene [78]	Graphene fiber	-	PF and *ZT* of 624 µW/mK^2^ and 2.76 × 10^−3^ respectively
PEDOT:PSS ^d^ doped with 5 wt.% DMSO ^e^ [79]	Polyester fabric	Solution coating	5 PEDOT:PSS coated strips generated 4.3 mV output at a Δ*T* = 75.2 K
p-type PEDOT:PSS, n-type MWCNT ^f^/PVP ^g^ [7]	PET ^h^ yarn	Solution coating	Maximum power output ≈ 7.4 nW for 38 TE legs
PEDOT:PSS/CNT ^i^ (p-type) yarn and PEI ^j^/CNT yarn (n-type) [6]	CNT yarns combined with PET yarns	Twisting with PET yarns for structural support	Maximum power output = 51.5 mW m^−2^
SWCNT ^k^/PANI ^l^ composite [80]	Polyester fabric	Dip coating	At Δ*T* = 75 K, power output = 47 nW
p-type SWCNT/DWCNT ^m^ and n-type PEI/SWCNT/DWCNT [81]	PTFE membrane	Vacuum filtration deposition	72 p-type and 72 n-type CNT films produced 465 mV at a temperature gradient of 49 K

^a^ Atomic layer deposition. ^b^ Molecular layer deposition. ^c^ Polyacrylonitrile. ^d^ poly(3,4-ethylenedioxythiophene) polystyrene sulfonate. ^e^ Dimethly sulfoxide. ^f^ Multiwalled Carbon Nanotubes. ^g^ Polyvinylpyrrolidone. ^h^ Polyethylene terephthalate. ^i^ Carbon Nanotube. ^j^ Polyethylenimine. ^k^ Singlewalled Carbon Nanotubes. ^l^ Polyaniline. ^m^ Doublewalled Carbon Nanotubes.

## Data Availability

Not applicable.
